# Systematic review of effectiveness of universal self-regulation-based interventions and their effects on distal health and social outcomes in children and adolescents: review protocol

**DOI:** 10.1186/s13643-017-0570-z

**Published:** 2017-08-29

**Authors:** Anuja Pandey, Daniel Hale, Anne-Lise Goddings, Sarah-Jayne Blakemore, Russell Viner

**Affiliations:** 1 UCL Great Ormond Street Institute of Child Health, Population, Policy and Practice Programme, 30 Guilford Street, London, WC1N 1EH UK; 2 Heriot-Watt University, Edinburgh, UK; 3 UCL Great Ormond Street Institute of Child Health, London, UK; 4 UCL Institute of Cognitive Neuroscience, London, UK

**Keywords:** Self-regulation, Interventions, Children, Adolescent

## Abstract

**Background:**

Growing evidence suggests that childhood and adolescence self-regulation contributes to multiple health, educational and social outcomes. Considering the potential impact of self-regulation skills on improved life chances in conjunction with evidence suggesting that self-regulation can be modified by interventions, there is a need to identify interventions which are most effective in improving childhood and adolescence self-regulation. The present systematic review was designed to determine the effectiveness of universal interventions focused on enhancing the self-regulation of children and adolescents. As secondary outcomes, we will also examine the effectiveness of such interventions on distal health and social outcomes.

**Methods:**

Eligible studies include randomised controlled trials (including cluster randomised trials) reporting on universal interventions designed to improve self-regulation in childhood and adolescence (age 0–19 years). The following databases will be searched for peer-reviewed publications using an iterative search strategy: Medline, PsycINFO, EMBASE, ERIC, CINAHL Plus, British Education Index, Child Development & Adolescent Studies and CENTRAL without applying language or date filters. Additionally, reference lists and citations of included studies will be searched for eligible studies. A 10% proportion of the total titles and abstracts will be randomly selected and screened independently by two reviewers (AP and DH). Results will be compared to ensure less than 5% discrepancy, followed by screening of all results by one reviewer (AP). Full-text review and data collection will be independently performed by two reviewers. Any discrepancies will be solved by mutual discussion, and if unresolved, a third reviewer (RV) will be consulted. Meta-analysis will be conducted to quantify trial effects, if the data is sufficiently homogenous to allow quantitative synthesis. Otherwise, results will be described narratively.

**Discussion:**

The evidence derived from the systematic review will strengthen the evidence base to inform planning of effective interventions targeting self-regulation skills in childhood and adolescence. This will benefit policy makers, academicians, researchers, health professionals, and also, young people who will benefit from policy and interventions informed by this review.

**Systematic review registration:**

CRD42016047661.

**Electronic supplementary material:**

The online version of this article (10.1186/s13643-017-0570-z) contains supplementary material, which is available to authorized users.

## Background

Self-regulation, also known as self-control, encompasses a range of competencies including capacity for controlling emotions, positive interactions with others, avoiding inappropriate or aggressive actions, and becoming a self-directed learner [1]. The cognitive components of self-regulation, referred to as executive function, includes the ability to direct attention, shift perspective, and adapt flexibly to changes (cognitive flexibility), retain information (working memory), and inhibit automatic or impulsive responses in order to achieve a goal such as problem solving (impulse control) [2, 3].

Evidence suggests that self-regulation skills can be a powerful predictor of positive health, educational, financial and social outcomes. Positive effects on a range of outcomes have been reported including school readiness, academic achievement, healthy behaviour, physical and mental health outcomes [4–7]. Conversely, poor self-regulation has been linked to adverse outcomes like health risk behaviours, psychiatric disorders, substance dependence, crime and unemployment [8–12]. Self-regulation is considered to be a malleable skill which can be shaped by environment, thus making it an important target for interventions [13, 14]. Children and adolescents are an important target group for such interventions considering the potential long-term benefits of positive health and social outcomes in this group. Though interventions to target self-regulation skills in childhood and adolescence are in their infancy, they have demonstrated potential for modification of self-regulation [15–17]. It is vital to recognise the nature of interventions that are most effective in improving self-regulation skills and thus impact outcomes in multiple domains.

No review has summarised the effectiveness of self-regulation interventions on health and social outcomes in children and adolescents, with previous systematic reviews restricted to specific populations or age-groups [18, 19]. Further, reviews have not examined the effects of interventions on distal health and social outcomes. With this background, we propose to conduct a systematic review with the objective of assessing the effectiveness of self-regulation-based interventions in improving self-regulation in children and adolescents. As a secondary objective, we also intend to study the effectiveness of such interventions in improving various health and social outcomes. We are interested in all potential effects of such interventions and shall include all reported outcomes of such interventions from eligible studies.

### Review question

What is the effectiveness of universal self-regulation-based interventions to improve self-regulation skills and distal health and social outcomes in children and adolescents?

## Methods/design

The review protocol is reported according to the Preferred Reporting Items for Systematic Review and Meta-Analysis Protocols (PRISMA-P) guidance, which can be found in Additional file 1.

### Eligibility criteria

#### Population

Our review will be restricted to studies on human participants. Studies will be eligible for inclusion if they report on children and/or adolescents (0–19 years). In cases where studies include participants beyond this age, the study will be included only if the median/mean age group of study participants falls within the 0–19 year range.

#### Intervention

In order to be included in the review, studies should explicitly state that it includes intervention designed to improve self-regulation. It may be the only intervention or a part of a package of multiple interventions. Under this criterion, self-regulation (or a related-term) must be mentioned in the title or key sentences describing the nature of the intervention.

Only universal interventions (interventions targeted to entire group of children and/or adolescents, not having or identified as having risk of any medical/behavioural disorder) intended to improve self-regulation shall be included for the review.

Interventions designed to improve self-regulation may vary by the type of activity (classroom-based activities, presentations or exercises), content, settings (school based, out of school, community based etc.) format (face to face or online), delivery methods (one to one or group), child based/parent based and intensity (in terms of length and frequency).

There will be no exclusion based on the type, format, settings and intensity of intervention.

#### Comparisons

We will include studies which compare self-regulation interventions with those receiving usual care, wait-listed groups, groups receiving no intervention or an intervention unrelated to self-regulation.

### Outcome

#### Primary outcome

Studies will be included if they report at least one child-/adolescent-based outcome measure related to self-regulation skills. This may include self-regulation skills as reported by teachers, educational administrators, study subjects (self-reported), parents, and/or researchers (subjective measures) as well as task performance-based measures indicated by objective measures.

#### Secondary outcome

We are interested in outcomes that are related to health and social parameters. However, we are also interested in understanding the different outcomes resulting from self-regulation skills and therefore our search strategy will not be limited by outcomes. The secondary outcomes may include, but might not be limited to health and social outcomes such as school readiness, academic achievement, physical health, mental health, employment, income and substance abuse.

#### Types of studies

Our review will be restricted to randomised (including cluster randomised) controlled trials of interventions.

#### Publication characteristics

Original research manuscripts published in peer-reviewed journals will be included. There will be no exclusion on the basis of study country; however, we will only include studies published in English. We shall not apply limits on the date of publication.

### Search strategy

We developed the search strategy after a number of initial scoping searches, with input from experts in the field. The strategy was further refined following a series of test searches and the discussion of the results among the review team. We designed our search strategy to include a list of terms, including controlled vocabulary terms for various databases to capture all variations within each of the three categories: population, intervention and study design). Since we are interested in all outcomes, no filters will be applied for outcomes. A published search filter (Cochrane 2008 Highly Sensitive Search Strategy) will be used for the section of the search strategy pertaining to study design [20]. We will search the following electronic databases: Medline, PsycINFO, Excerpta Medica Database (EMBASE) via OVID; Education Resources Information Center (ERIC), Cumulative Index to Nursing and Allied Health Literature (CINAHL), PLUS, British Education Index, Child Development & Adolescent Studies via EBSCO and Cochrane Controlled Trials Register (CENTRAL) without applying language or date filters. The detailed search strategy for each database is available in Additional file 2. A preliminary search in July 2016 yielded the following results: (Medline, *n* = 4538; PsycINFO, *n* = 5703; EMBASE, *n* = 5425; ERIC, *n* = 1592; CINAHL PLUS = 1112; British Education Index, *n* = 88; Child Development and adolescent studies, *n* = 707; and Cochrane, *n* = 2432). We will import the search results into EPPI-Reviewer 4 software, which will be used throughout the review to store and manage records. An updated search will be carried out, and additionally, we shall also search reference lists and citations of included articles for eligible studies.

### Study selection process

Titles and abstracts will be screened to identify studies eligible for full-text review based on predefined eligibility criteria. A 10% proportion of the total titles and abstracts will be randomly selected and screened independently by two reviewers (AP and DH), followed by screening of all results by one reviewer (AP). Screening results by both reviewers for subsample screening will be compared to ensure if there is agreement on at least 95% titles and abstracts (Kappa more than 0.7). If there is lack of agreement on more than 5%, the entire sample will be rescreened by both reviewers following a training on review protocol. Full-text papers of studies fulfilling eligibility criteria on title and abstract screening will be obtained. Full-text articles of eligible studies will be assessed independently by two reviewers (AP and DH) against the study inclusion criteria. Articles that do not fulfil the eligibility criteria would be allotted an exclusion justification code and excluded. If there are discrepancies regarding study inclusion among the reviewers, it will be solved by consensus or by a third reviewer (RV), if necessary. If the study details are not sufficient to determine eligibility, corresponding authors will be contacted for further details. If such details are not available, the study will be deemed ineligible. Review authors will not be blinded to author name, institution or journal title.

### Data extraction and quality assessment

Data will be extracted using pre-piloted data extraction forms (see Table 1). Two reviewers (AP and DH) will independently extract data regarding the study participants, characteristics of intervention and its evaluation and do quality assessment. Any discrepancy in data extraction will be resolved by mutual consensus.

**Table 1:** Data extraction form

Characteristics of included studies
Study ID (author name and year) Author email Country Study design (RCT/cluster RCT)
Participants
Total number Age group (range/average age) Setting (including location and social context) Method of recruitment of participants Clusters (if applicable, no., type, no. people per cluster, no of sites) Baseline imbalances Withdrawals and exclusions (if not provided below by outcome) Sex (male to female ratio) Race/ethnicity Any special group/conditions/relevant social demographics
Intervention/control group (separate section for each group)
Unit of allocation (group/individual) No. randomised to group (specify whether no. people or clusters; if clusters, number of sites) Intervention description (content, components) Timing (frequency, duration of each episode) Delivery (mechanism, medium, intensity) Providers (e.g. no., profession, training, ethnicity etc. if relevant) Co-interventions Integrity of delivery Compliance
Outcomes (separate section for each outcome)
Outcome name Time points measured Any outcome definition/scale Person measuring/reporting Unit of measurement Is outcome tool validated? Effect measures: point estimate, confidence intervals
Additional comments:

Quality assessment of included studies will be done by assessing the studies with regards to selection bias, study design, confounders, blinding, data collection methods, withdrawals and drop outs, intervention integrity and analyses using the Effective Public Health Practice Project (EPHPP) Quality Assessment Tool For Quantitative Studies [21], which is a validated tool for quality assessment.

Attempts will be made to resolve disagreements on quality assessment by two reviewers through discussion and consensus. If there is no agreement, a third reviewer (RV) will be consulted.

### Data synthesis and analysis

Detailed information about included studies will be extracted independently by two review authors (AP and DH) regarding the characteristics of study participants, interventions and its evaluation. Data will be assessed for intervention effects in terms of change in self-regulation and other health and social outcomes. If there are multiple points of measurements, then intervention effects will be reported as short term (<1 year) or long term (>1 year). If there are multiple follow-up assessments, the data from the final trial endpoint fulfilling age eligibility will be used.

Quantitative data would be extracted and wherever sufficient data is available, odds ratio (for categorical outcome data) or standardised mean differences/Cohen’s *d* (for continuous data) and their 95% confidence intervals will be calculated from the extracted data from each included randomised controlled trial. Multiple papers of the same study would be coded as related studies. If needed and deemed appropriate, data will be combined as a single study for inclusion in meta-analysis.

Meta-analysis will be performed using random effects model if the data is sufficiently homogenous. Separate meta-analysis will be conducted for each outcome if enough information is available to conduct such analysis. To assess heterogeneity, we will calculate *I* ^2^ statistic and visually inspect forest plots. If heterogeneity exists, the sources of heterogeneity will be investigated and sub-group analysis will be conducted. If quantitative synthesis is not possible, a narrative review will be presented.

#### Issues of clustering

It is likely that in some of the cluster randomised trials, effects of clustering might not be adjusted in the original studies. In such situations, intra-class correlations will be requested from corresponding authors. If such information is not available, estimates will be obtained from similar studies and generic inverse variance approach would be used to combine their results.

## Discussion

This review will aid in building an evidence base for the effectiveness of self-regulation interventions in children and adolescents. Self-regulation has sparked policy interest internationally, as a part of ongoing search to define abilities that predict success and better life chances. Identifying interventions that demonstrate a potential for modification of self-regulation in childhood and adolescence will provide an opportunity to better inform the development of interventions for this group to potentially promote health and social outcomes in child and adolescent populations. The review will be useful to policy makers, academicians, researchers and health professionals who are interested in such interventions. The review will also identify any gaps in the existing evidence to provide direction for future research.

Given the complex nature of the construct of self-regulation and the lack of a uniform definition, we expect that there will be inconsistency in the studies in defining and measuring self-regulation. We will take this variability into account when combining results and interpreting them. Also, issues of unit of analysis will be taken into account while combining data from randomised and cluster randomised studies.

## Additional files

Additional file 1: Completed PRISMA-P Checklist (DOCX 29 kb)

Additional file 2: Electronic Search Strategy for various databases searched (DOCX 25 kb)

## Abbreviations

CENTRAL: Cochrane central register of controlled trials
CINAHL: Cumulative index to nursing and allied health literature
EMBASE: Excerpta Medica Database
ERIC: Education Resources Information Center
PRISMA-P: Preferred Reporting Items for Systematic Review and Meta-Analysis Protocols
RCT: Randomised controlled trial
