# Supplementary material for: Systematic review of effectiveness of universal self-regulation-based interventions and their effects on distal health and social outcomes in children and adolescents: review protocol
Source: Syst Rev. 2017 Aug 29;6:175. doi: 10.1186/s13643-017-0570-z (PMC5576387; doi:10.1186/s13643-017-0570-z)
Supplement: Supplementary file 2 — Electronic Search Strategy for various databases searched (DOCX 25 kb) [file 13643_2017_570_MOESM2_ESM.docx]

**ADDITIONAL FILE 1: ELECTRONIC SEARCH STRATEGY**

| **I**. | **MEDLINE® via Ovid MEDLINE 1946 to July 14,2016** |
| --- | --- |
| 1 | exp Child/ |
| 2 | children.mp. |
| 3 | childhood.mp. |
| 4 | Child, Preschool/ |
| 5 | preschool.mp. |
| 6 | Schools/ |
| 7 | school*.mp. |
| 8 | exp Adolescent/ |
| 9 | adolesc*.mp. |
| 10 | teen*.mp. |
| 11 | youth.mp. |
| 12 | kindergarten.mp. |
| 13 | class.mp. |
| 14 | classroom.mp. |
| 15 | juvenile.mp. |
| 16 | 1 or 2 or 3 or 4 or 5 or 6 or 7 or 8 or 9 or 10 or 11 or 12 or 13 or 14 or 15 |
| 17 | exp Self-Control/ |
| 18 | self control.mp. |
| 19 | Self regulat*.mp. |
| 20 | self manag*.mp. |
| 21 | Executive Function/ |
| 22 | executive function*.mp. |
| 23 | Metacognition/ |
| 24 | metacognit*.mp. |
| 25 | effortful control.mp. |
| 26 | (attention adj3 control).mp. |
| 27 | 17 or 18 or 19 or 20 or 21 or 22 or 23 or 24 or 25 or 26 |
| 28 | randomized controlled trial.pt. |
| 29 | controlled clinical trial.pt. |
| 30 | randomized.ti,ab. |
| 31 | Placebo.ti,ab. |
| 32 | Drug Therapy/ |
| 33 | randomly.ti,ab. |
| 34 | trial.ti,ab. |
| 35 | groups.ti,ab. |
| 36 | 28 or 29 or 30 or 31 or 32 or 33 or 34 or 35 |
| 37 | Animals/ |
| 38 | Humans/ |
| 39 | 37 not 38 |
| 40 | 36 not 39 |
| 41 | 16 and 27 and 40 |

| **II.** | **PSYCINFO (Via Ovid) dt.14/07/16** |
| --- | --- |
| 35 | 15 AND 28 AND 34 |
| 34 | 29 or 30 or 31 or 32 or 33 |
| 33 | group.mp. |
| 32 | exp PLACEBO/ |
| 31 | exp Intervention/ or exp Clinical Trials/ or exp Treatment Effectiveness Evaluation/ or exp Treatment Outcomes/ |
| 30 | trial*.ti,ab,hw,id. |
| 29 | random*.ti,ab,hw,id. |
| 28 | 16 or 17 or 18 or 19 or 20 or 21 or 22 or 23 or 24 or 25 or 26 or 27 |
| 27 | metacognit*.mp. |
| 26 | (attention adj3 control).mp. |
| 25 | effortful control.mp. |
| 24 | exp METACOGNITION/ |
| 23 | executive function*.mp. |
| 22 | exp Executive Function/ |
| 21 | self manag*.mp. |
| 20 | exp Self-Management/ |
| 19 | exp Self-Regulation/ |
| 18 | Self regulat*.mp. |
| 17 | self control.mp. |
| 16 | exp Self-Control/ |
| 15 | 1 or 2 or 3 or 4 or 5 or 6 or 7 or 8 or 9 or 10 or 11 or 12 or 13 or 14 |
| 14 | juvenile.mp. |
| 13 | classroom.mp. |
| 12 | class.mp. |
| 11 | school*.mp. |
| 10 | exp JUNIOR HIGH SCHOOLS/ or exp NURSERY SCHOOLS/ or exp HIGH SCHOOLS/ or exp BOARDING SCHOOLS/ or exp MIDDLE SCHOOLS/ or exp SCHOOLS/ or exp ELEMENTARY SCHOOLS/ |
| 9 | youth.mp. |
| 8 | teen*.mp. |
| 7 | adolesc*.mp. |
| 6 | adolescent.mp. |
| 5 | preschool.mp. |
| 4 | exp PRESCHOOL STUDENTS/ |
| 3 | childhood.mp. |
| 2 | children.mp. |
| 1 | child.mp. |

| **III.** | **EMBASE Via Ovid dt.15/07/16** |
| --- | --- |
| 40 | 16 and 26 and 39 |
| 39 | 35 not 38 |
| 38 | 36 not 37 |
| 37 | humans/ |
| 36 | animals/ |
| 35 | 27 or 28 or 29 or 30 or 31 or 32 or 33 or 34 |
| 34 | groups.ti,ab. |
| 33 | trial.ti,ab. |
| 32 | randomly.ti,ab. |
| 31 | drug therapy/ |
| 30 | placebo.ti,ab. |
| 29 | randomized.ti,ab. |
| 28 | controlled clinical trial/ |
| 27 | randomized controlled trial/ |
| 26 | 17 or 18 or 19 or 20 or 21 or 22 or 23 or 24 or 25 |
| 25 | executive function*.mp. |
| 24 | (attention adj3 control).mp. |
| 23 | effortful control.mp. |
| 22 | metacognit*.mp. |
| 21 | executive function.mp. or executive function/ |
| 20 | self manag*.mp. |
| 19 | Self regulat*.mp. |
| 18 | self control.mp. |
| 17 | self control/ |
| 16 | 1 or 2 or 3 or 4 or 5 or 6 or 7 or 8 or 9 or 10 or 11 or 12 or 13 or 14 or 15 |
| 15 | classroom.mp. |
| 14 | class.mp. |
| 13 | kindergarten/ or nursery/ |
| 12 | juvenile/ |
| 11 | youth.mp. |
| 10 | teen*.mp. |
| 9 | adolescence/ |
| 8 | adolescent/ |
| 7 | adolesc*.mp. |
| 6 | school*.mp. |
| 5 | nursery school/ or school/ or middle school/ or high school/ or primary school/ or high school student/ or middle school student/ |
| 4 | preschool.mp. |
| 3 | children.mp. |
| 2 | childhood/ |
| 1 | child/ |

| **IV. Cochrane Central Search Strategy dt. 15/07/16** |
| --- |
| #1 MeSH descriptor: [Child, Preschool] this term only  #2 children  #3 child  #4 childhood  #5 school*  #6 kindergarten  #7 teen*  #8 adolesc*  #9 youth  #10 class  #11 classroom  #12 juvenile  #13 preschool  #14 #1 or #2 or #3 or #4 or #5 or #6 or #7 or #8 or #9 or #10 or #11 or #12 or #13  #15 "self control"  #16 "self regulat*"  #17 "self manag*"  #18 "executive function*"  #19 "effortful control"  #20 "attention control"  #21 metacognit*  #22 #15 or #16 or #17 or #18 or #19 or #20 or #21  #23 #14 and #22  ……….[Control results by: Clinical Trials] |

| **V.** | **CINAHL PLUS Via EBSCO (15/07/16)** |
| --- | --- |
| S35 | S13 AND S21 AND S34 |
| S34 | S30 NOT S33 |
| S33 | animals NOT humans |
| S32 | humans or people |
| S31 | animals |
| S30 | S22 OR S23 OR S24 OR S25 OR S26 OR S27 OR S28 OR S29 |
| S29 | AB groups |
| S28 | AB trial |
| S27 | AB randomly |
| S26 | AB randomized |
| S25 | AB placebo |
| S24 | drug therapy |
| S23 | clinical trials |
| S22 | randomized controlled trials |
| S21 | S14 OR S15 OR S16 OR S17 OR S18 OR S19 OR S20 |
| S20 | AB "attention adj3 control" |
| S19 | AB "effortful control" |
| S18 | AB "self manag*" |
| S17 | AB "self regulat*" |
| S16 | AB "self control" |
| S15 | AB "executive function*" |
| S14 | AB metacognit* |
| S13 | S1 OR S2 OR S3 OR S4 OR S5 OR S6 OR S7 OR S8 OR S9 OR S10 OR S11 OR S12 |
| S12 | AB classroom |
| S11 | AB class |
| S10 | AB kindergarten |
| S9 | AB juvenile |
| S8 | AB teen* |
| S7 | AB youth |
| S6 | AB school* |
| S5 | AB adolesc* |
| S4 | AB preschool |
| S3 | AB childhood |
| S2 | AB children |
| S1 | AB child |

| **VI.** | **Child Development & Adolescent Studies Via EBSCO (15/07/16)** |
| --- | --- |
| S35 | S13 AND S21 AND S34 |
| S34 | S30 NOT S33 |
| S33 | animals NOT humans |
| S32 | humans or people |
| S31 | animals |
| S30 | S22 OR S23 OR S24 OR S25 OR S26 OR S27 OR S28 OR S29 |
| S29 | AB groups |
| S28 | AB trial |
| S27 | AB randomly |
| S26 | AB randomized |
| S25 | AB placebo |
| S24 | drug therapy |
| S23 | clinical trials |
| S22 | randomized controlled trials |
| S21 | S14 OR S15 OR S16 OR S17 OR S18 OR S19 OR S20 |
| S20 | AB "attention adj3 control" |
| S19 | AB "effortful control" |
| S18 | AB "self manag*" |
| S17 | AB "self regulat*" |
| S16 | AB "self control" |
| S15 | AB "executive function*" |
| S14 | AB metacognit* |
| S13 | S1 OR S2 OR S3 OR S4 OR S5 OR S6 OR S7 OR S8 OR S9 OR S10 OR S11 OR S12 |
| S12 | AB classroom |
| S11 | AB class |
| S10 | AB kindergarten |
| S9 | AB juvenile |
| S8 | AB teen* |
| S7 | AB youth |
| S6 | AB school* |
| S5 | AB adolesc* |
| S4 | AB preschool |
| S3 | AB childhood |
| S2 | AB children |
| S1 | AB child |

| **VII.** | **British Education Index Via EBSCO (15/07/16)** |
| --- | --- |
| S35 | S13 AND S21 AND S34 |
| S34 | S30 NOT S33 |
| S33 | animals NOT humans |
| S32 | humans or people |
| S31 | animals |
| S30 | S22 OR S23 OR S24 OR S25 OR S26 OR S27 OR S28 OR S29 |
| S29 | AB groups |
| S28 | AB trial |
| S27 | AB randomly |
| S26 | AB randomized |
| S25 | AB placebo |
| S24 | drug therapy |
| S23 | clinical trials |
| S22 | randomized controlled trials |
| S21 | S14 OR S15 OR S16 OR S17 OR S18 OR S19 OR S20 |
| S20 | AB "attention adj3 control" |
| S19 | AB "effortful control" |
| S18 | AB "self manag*" |
| S17 | AB "self regulat*" |
| S16 | AB "self control" |
| S15 | AB "executive function*" |
| S14 | AB metacognit* |
| S13 | S1 OR S2 OR S3 OR S4 OR S5 OR S6 OR S7 OR S8 OR S9 OR S10 OR S11 OR S12 |
| S12 | AB classroom |
| S11 | AB class |
| S10 | AB kindergarten |
| S9 | AB juvenile |
| S8 | AB teen* |
| S7 | AB youth |
| S6 | AB school* |
| S5 | AB adolesc* |
| S4 | AB preschool |
| S3 | AB childhood |
| S2 | AB children |
| S1 | AB child |

| **VIII.** | **ERIC Via EBSCO (15/07/16)** |
| --- | --- |
| S35 | S13 AND S21 AND S34 |
| S34 | S30 NOT S33 |
| S33 | animals NOT humans |
| S32 | humans or people |
| S31 | animals |
| S30 | S22 OR S23 OR S24 OR S25 OR S26 OR S27 OR S28 OR S29 |
| S29 | AB groups |
| S28 | AB trial |
| S27 | AB randomly |
| S26 | AB randomized |
| S25 | AB placebo |
| S24 | drug therapy |
| S23 | clinical trials |
| S22 | randomized controlled trials |
| S21 | S14 OR S15 OR S16 OR S17 OR S18 OR S19 OR S20 |
| S20 | AB attention adj3 control |
| S19 | AB "effortful control" |
| S18 | AB "self manag*" |
| S17 | AB "self regulat*" |
| S16 | AB "self control" |
| S15 | AB "executive function*" |
| S14 | AB metacognit* |
| S13 | S1 OR S2 OR S3 OR S4 OR S5 OR S6 OR S7 OR S8 OR S9 OR S10 OR S11 OR S12 |
| S12 | AB classroom |
| S11 | AB class |
| S10 | AB kindergarten |
| S9 | AB juvenile |
| S8 | AB teen* |
| S7 | AB youth |
| S6 | AB school* |
| S5 | AB adolesc* |
| S4 | AB preschool |
| S3 | AB childhood |
| S2 | AB children |
| S1 | AB child |
